# Human Papilloma Virus Infection and Vaginal Microbiome Profiles in Pre-menopausal Women: A Cross-Sectional Study

**DOI:** 10.7759/cureus.89872

**Published:** 2025-08-12

**Authors:** Smriti Shende, Rashmita Das, Suvarna Joshi, Sushma Yanamandra, Nyabom Taji, Rajesh P Karyakarte

**Affiliations:** 1 Microbiology, B. J. Government Medical College & Sassoon General Hospitals, Pune, IND; 2 Microbiology, Tomo Riba Institute of Health and Medical Sciences, Itanagar, IND

**Keywords:** 16s rrna gene sequencing analysis, community state types (csts), hpv infection, pap smear study in cancer cervix, pre-menopausal women, vaginal microbiome

## Abstract

Background: Cervical cancer is a common cancer among women worldwide, especially in low- and middle-income countries. Persistent infection with high-risk human papillomavirus (HR-HPV) largely drives the development of cervical cancer. While *Lactobacillus*-dominant communities are considered protective, dysbiosis, marked by reduced *Lactobacilli* and increased anaerobic diversity (community state type (CST)-IV), may promote viral persistence. This study aims to assess HR-HPV prevalence and compare vaginal microbiome profiles in women with suspected HPV infection and healthy controls.

Material and methods: Vaginal swabs were collected from pre-menopausal women with clinically suspected HPV infection and healthy controls. Samples underwent HR-HPV detection using the Truenat® HPV-HR RT-PCR (real-time polymerase chain reaction) assay (Molbio Diagnostics Limited, Goa, India). Vaginal microbiome profiling was performed using 16S rRNA gene amplicon sequencing on the GridION platform (Oxford Nanopore Technologies plc, Oxford, United Kingdom). Taxonomic classification was carried out using the EPI2ME 16S workflow with Kraken2 (Johns Hopkins University, Baltimore, Maryland, United States), and CSTs were assigned using the VALENCIA (VAginaL community state typE Nearest CentroId clAssifier) algorithm. Statistical analyses and microbial community comparisons were performed using MicrobiomeAnalyst (Xia Lab, Sainte-Anne-de-Bellevue, Quebec, Canada).

Results: A total of 86 clinically suspected HPV cases and 63 healthy pre-menopausal controls were enrolled. Overall, high-risk HPV (HPV 16/31 and HPV-18/45) was detected in 11.6% (10/86) of cases, with no positivity in the control group. Vaginal microbiome profiling revealed significantly higher alpha diversity in women with inflammatory cytology compared to healthy controls, and increased Shannon and Simpson diversity indices in HPV-positive and inflammatory groups. Beta diversity analysis showed distinct microbial clustering between all groups. Taxonomic analysis demonstrated a predominance of *Lactobacillus* spp. in healthy individuals, particularly *Lactobacillus crispatus* and *Lactobacillus iners*, whereas disease groups showed increased abundance of *Pseudomonas*, *Rheinheimera*, and *Agrobacterium*. CST-I was more common in healthy controls (7/17, 41.2%), while CST-IV-linked to dysbiosis-was predominant among suspected cases (9/21, 47.6%). Linear discriminant analysis effect size (LEfSe) analysis identified *Lactobacillus*, *Anaerococcus*, and *Dialister* as key genera in healthy individuals, whereas *Pseudomonas*, *Rhizobium*, and *Rheinheimera* were enriched in HPV-positive and inflammatory smear groups, highlighting potential microbial biomarkers of vaginal dysbiosis.

Conclusion: These findings underscore the importance of vaginal microbiome composition in cervical health and support further investigation into microbial biomarkers for early detection and targeted interventions in HPV-associated disease.

## Introduction

Cervical cancer ranks as the fourth most prevalent cancer among women globally, with an estimated 660,000 new diagnoses and approximately 350,000 deaths reported in 2022. Notably, 94% of these deaths occurred in low- and middle-income countries [1]. Human papillomavirus (HPV), a circular, double-stranded, non-enveloped DNA virus belonging to the Papovaviridae family, is implicated in the vast majority of cervical squamous cell carcinoma cases, accounting for approximately 99.7%. Over 200 HPV genotypes have been identified, among which at least 15 are strongly associated with the development of cervical cancer. Genital HPV types are broadly classified into high-risk (oncogenic) and low-risk (non-oncogenic) categories based on their potential to cause cervical cancer and its precursor lesions. High-risk HPV (HR-HPV) types, including 16, 18, 31, 33, 35, 39, 45, 51, 52, 56, 58, 59, 68, 73, and 82, play a central role in the pathogenesis of cervical cancer [2]. While most HPV infections are transient and cleared by the immune system, persistent HR-HPV infections may progress to cervical cancer and other anogenital malignancies [3].

While persistent infection with oncogenic HPV is essential for cervical cancer development, it alone is insufficient to induce malignancy. Several cofactors influence HPV persistence and the progression toward cancer, including immunosuppression (such as HIV infection), smoking, extended use of hormonal contraceptives, and recent evidence indicating alterations in vaginal microbiota composition [4]. Advancements in next-generation sequencing (NGS) technologies have significantly enhanced our capacity to study the microbial communities at detailed phylogenetic and taxonomic levels, enabling a deeper investigation into how vaginal microbiome alterations may contribute to HPV persistence and cervical disease. Molecular profiling of microbial communities has provided critical insights into host-microbe interactions and their implications in various disease states [5,6].

The vaginal microbiome represents a dynamic microecosystem that continually fluctuates throughout a woman's lifetime. It comprises stratified squamous, nonkeratinized epithelium covered by cervicovaginal secretions, creating a predominantly anaerobic environment due to limited vascularization and nutrient diffusion from submucosal tissues. This unique habitat supports a complex microbial community that coexists symbiotically with the host, forming the vaginal microbiome [7]. The structure of the vaginal microbiome is influenced by multiple factors such as age, ethnicity, genetics, lifestyle, diet, hygiene, infections, antibiotic use, sexual behaviour, and estrogen levels [6].

Numerous studies have explored the connection between vaginal microbiota composition and HPV infection, emphasizing its potential role in cervical precancerous lesion development and cancer progression [8]. A healthy vaginal microbiome (eubiosis) typically exhibits* Lactobacillus* dominance, contributing to vaginal health by producing lactic acid, lowering pH, and preventing pathogen growth. Conversely, dysbiosis involves reduced *Lactobacillus* levels, increased microbial diversity, and anaerobic proliferation, creating conditions more conducive to HPV persistence [9]. Therefore, based on the dominance of specific *Lactobacillus* species, vaginal microbiomes are commonly categorized into community state types (CSTs). The presence of CSTs I, II, III, and V is characterized by *Lactobacillus crispatus*, *Lactobacillus gasseri*, *Lactobacillus iners*, and *Lactobacillus jensenii*, respectively. At the same time, CST IV is marked by diverse anaerobic species such as *Gardnerella vaginalis* and *Prevotella* species [8,6]. The distribution of these CSTs differs according to the disease state, notably with CST-I and CST-V becoming less common and CST-IV increasingly predominant as the disease progresses [7].

Considering these findings, the present study focuses on assessing the prevalence of HR-HPV among women with suspected cervical cancer and comparing it with healthy controls. Additionally, the study aims to characterize and compare the vaginal microbiome profiles in both groups. By integrating HPV molecular detection with microbiome profiling, this study endeavours to identify microbial patterns that may influence HPV infection and persistence.

## Materials and methods

This study was conducted at the Department of Microbiology, B. J. (Byramjee Jeejeebhoy) Government Medical College (BJGMC), Pune, Maharashtra, India, from January 2021 to June 2022. The study was approved by the Institutional Ethics Committee, BJGMC & Sassoon General Hospitals (approval number: BJGMC/IEC/Pharmac/D-0121033-033).

Eligibility criteria

The study enrolled pre-menopausal women who were clinically suspected of HPV infection based on the presence of symptoms such as persistent vaginal discharge, postcoital bleeding, intermenstrual bleeding, or visible cervical lesions on per speculum examination. Women who had a prior history of sexually transmitted infections (STIs), hormonal therapy in the past six months, and had received the HPV vaccine were excluded from the study. Additionally, pre-menopausal women with no clinical symptoms suggestive of genital infections, cervical pathology, or other gynaecological complaints and no history of multiple sexual partners were included as healthy controls.

Sample collection

Following consent, vaginal swab samples were collected from 86 clinically suspected HPV cases and 63 healthy pre-menopausal controls in the Gynaecology Outpatient Department (OPD). Swabs were obtained from the posterior and lateral fornices of the vagina using sterile nylon flocked swabs, which were then placed in sterile viral transport media (VTM) tubes and transported to the Microbiology Laboratory. Each sample was divided into two aliquots: one aliquot for HPV detection and genotyping, and the second aliquot for 16S rRNA gene-based sequencing to minimize contamination and avoid freeze-thaw cycles. All samples were stored at −80°C until further processing. Papanicolaou (Pap) smears were collected and sent to the Pathology Laboratory for cytological examination.

Pap smear examination

Pap smear slides were stained using the Papanicolaou stain, and cytological evaluation was performed following the Bethesda system for reporting cervical cytology [10].

HPV detection and genotyping

Vaginal swab samples were processed using the Trueprep® Universal Cartridge-Based Sample Prep Device (Molbio Diagnostics Limited, Goa, India). HPV detection and genotyping were carried out using the Truenat® HPV-HR kit on the Truelab® Real-Time micro-Analyzer (Molbio Diagnostics Limited), targeting HR-HPV types 16/31 and 18/45.

16S rRNA gene-based amplicon sequencing

DNA Extraction and Quantification

Bacterial DNA was extracted from 22 HPV-suspected vaginal samples and 23 healthy individuals using the MagMAX™ Viral/Pathogen Nucleic Acid Isolation Kit (Applied Biosystems™; Thermo Fisher Scientific Inc., Waltham, Massachusetts, United States) and the Insta NX® Mag 96 Automated Nucleic Acid Extraction System (HiMedia Laboratories Private Limited, Mumbai, Maharashtra, India). HPV-suspected samples included those that were either HPV polymerase chain reaction (PCR) positive or had abnormal Pap smear cytology. Healthy individuals were randomly selected from participants who were HPV PCR negative and had normal Pap smear cytology. DNA concentration was measured using the Qubit dsDNA High Sensitivity Assay Kit on a Qubit 2.0 Fluorometer (Thermo Fisher Scientific Inc.). Extracted DNA was stored at −80°C until further analysis.

Library Preparation and 16S rRNA Sequencing

Library preparation and sequencing were conducted in two phases, beginning with 22 samples from clinically suspected HPV cases in the first phase (Run 1), followed by 23 samples from healthy individuals in the second phase (Run 2). Libraries for 16S rRNA (16S rRNA stands for 16S ribosomal ribonucleic acid (rRNA), where S (Svedberg) is a unit of measurement (sedimentation rate)) amplicon sequencing were prepared using the 16S Barcoding Kit 1-24 (SQK-16S024, version 16S_9086_v1_revM_14Aug2019) (Oxford Nanopore Technologies plc, Oxford, United Kingdom) following the manufacturer's protocol. Briefly, 10 nanograms of genomic DNA were used for 16S rRNA gene amplification with barcoded primers provided in the kit. Barcoded samples were quantified, pooled, and purified post-PCR amplification using the AMPure XP beads (Beckman Coulter, Brea, California, United States). Rapid sequencing adapters were attached to the purified DNA fragments. The prepared library was loaded onto primed FLO-MIN106 R9.4.1 sequencing flow cells using the Flow Cell Priming Kit EXP-FLP001 (Oxford Nanopore Technologies, Oxford, UK). Sequencing was performed on the GridION platform (Oxford Nanopore Technologies plc, Oxford, United Kingdom) following the manufacturer's standard protocols.

Data analysis workflow

Live basecalling and demultiplexing were performed using MinKNOW GUI software (Oxford Nanopore Technologies plc) to generate demultiplexed FASTQ files. The basecalling was performed with the Fast-basecalling model, using Barcode Kit SQK-16S024, with the trim_barcodes option enabled. Quality filtering was applied to retain only pass reads with a minimum quality score of 7.

The FASTQ files were processed using the wf-16S nextflow workflow (version 1.4.0) in EPI2ME Labs (https://epi2me.nanoporetech.com/) for bacterial taxonomy assignment. Briefly, per-sample FASTQ files were concatenated using fastcat (version 0.20.0). Taxonomic classification was performed using Kraken2 version 2.1.2 (Johns Hopkins University, Baltimore, Maryland, United States) with the National Center for Biotechnology Information (NCBI) 16s and 18s reference databases. Default filtering parameters, including reads shorter than 800 base pairs and longer than 2000 base pairs, were applied during the analysis. Additionally, taxa with an abundance threshold of ≤1 were removed to eliminate low-confidence classifications.

To classify the samples into distinct CSTs, the VALENCIA (VAginaL community state typE Nearest CentroId clAssifier) algorithm [11] was used. VALENCIA is a supervised nearest centroid-based classifier that assigns samples to established CSTs based on the composition of vaginal microbial communities.

Statistical analysis

Quantitative data were represented as percentages and proportions and were analyzed using mean and standard deviation (SD). Differences between the two means were assessed using the Z-test to determine statistical significance. For qualitative variables, comparisons between groups were performed using the Chi-square or Fisher's exact test, depending on the data distribution. A p-value of less than 0.05 was considered indicative of statistical significance.

Raw 16S rRNA gene sequencing data obtained using the EPI2ME 16S workflow were processed and analyzed using the MicrobiomeAnalyst platform (Xia Lab, Sainte-Anne-de-Bellevue, Quebec, Canada) [12]. An operational taxonomic unit (OTU) abundance table generated from taxonomic classification against the NCBI 16S/18S database was used as input. To reduce noise and improve the reliability of downstream analyses, low-quality features were removed through both abundance- and variance-based filtering. Briefly, taxa present in only one sample and those constant across all samples were removed. Further, taxa with a minimum count of four and prevalence in less than 20% of samples, along with those showing variance below 10% based on interquantile range (IQR), were excluded. Following data rarefaction, data normalization was performed using the Total Sum Scaling (TSS) method. All diversity analysis, including alpha and beta diversity, taxonomic composition and abundance, and core microbiome analyses, were conducted on the filtered and normalized dataset. Alpha diversity indices and taxonomic distribution were calculated with rarefied reads sampled to even depth. For beta diversity analysis, Bray-Curtis distances were calculated from the table of taxa counts. Principal coordinate analysis (PCoA) was performed using a Bray-Curtis similarity matrix at the phylum, genus, and species level datasets. Permutational analysis of variance (PERMANOVA) analysis was performed between the study groups to assess the difference in beta diversity.

## Results

Demographic characteristics of the study population

A total of 86 clinically suspected cases of HPV infection and 63 healthy pre-menopausal controls were included in the study. The demographic characteristics of the study population are summarized in Table 1. The mean age of participants in the case group was 37.4 ± 5.84 years, while in the control group, it was 29.2 ± 2.69 years. The majority of participants in the case group (85/86, 98.84%) and more than half of the control group (35/63, 55.56%) were married. Regarding parity, 73 out of 86 (84.88%) cases were multiparous, whereas 49 out of 63 (77.78%) controls were nulliparous. A history of contraceptive use was absent in most participants, with 81 (94.19%) cases and 59 (93.65%) of controls reporting no use of contraception. Most cases (79/86, 91.86%) and controls (42/63, 66.67%) reported having regular menstrual cycles. Among the cases, the most common presenting symptom was abdominal pain (71/86, 82.56%), followed by vaginal discharge (35/86, 40.70%), and both burning micturition and heavy menstrual flow were reported by 26 out of 86 (30.23%) participants.

**Table 1:** Demographic characteristics of the study participants

Demographic Characteristics	Cases (n=86), n (%)	Controls (n=63), n (%)
Age groups (in years)		
20-29	11 (12.79%)	11 (17.46%)
30-39	31 (36.05%)	25 (39.68%)
40-49	44 (51.16%)	27 (42.86%)
Marital status		
Married	85 (98.84%)	35 (55.56%)
Unmarried	01 (1.16%)	28 (44.44%)
Parity		
Nulliparous	02 (2.33%)	49 (77.78%)
Primiparous	11 (12.79%)	06 (9.52%)
Multiparous	73 (84.88%)	08 (12.70%)
History of use of contraception		
Present	05 (5.81%)	04 (6.35%)
i. Intrauterine Device (Copper-T)	05 (100%)	0
ii. Barrier Method	0	04 (100%)
Absent	81 (94.19%)	59 (93.65%)
Regularity of menstrual cycles		
Regular	79 (91.86%)	42 (66.67%)
Irregular	07 (8.14%)	21 (33.33%)
Presenting symptoms		
Burning micturition	26 (30.23%)	0
Heavy flow during menstruation	26 (30.23%)	0
Pain in abdomen	71 (82.56%)	0
Vaginal discharge	35 (40.70%)	0

Pap smear findings of clinically suspected HPV cases and healthy controls

Most cases (44/68, 64.71%) and controls (63/63, 100%) had normal Pap smear findings. Additionally, 22 out of 68 (32.35%) cases showed inflammatory cervical smears, and one (1.47%) each showed mild dysplasia and carcinoma in situ (Table 2).

**Table 2:** Pap smear findings of clinically suspected HPV cases and healthy controls *Pap smear data was unavailable for 18 of the 86 cases

Pap Smear Findings	Cases (n=68*), n (%)	Controls (n=63), n (%)
Normal Cytology	44 (64.71%)	63 (100%)
Inflammatory Cytology	22 (32.35%)	0
Mild Dysplasia	01 (1.47%)	0
Carcinoma In-situ	01 (1.47%)	0

HPV reverse transcription (RT)-PCR findings among clinically suspected HPV cases and healthy controls

The overall prevalence of HPV infection in the study population was 6.71% (10/149). All healthy controls tested negative for HPV infection by RT-PCR, while 11.63% (10/86) of the clinically suspected cases were HPV positive. Among the positive cases, three cases (30%) were positive for HPV genotype 16/31, and seven cases (70%) were positive for HPV genotype 18/45 (Table 3).

**Table 3:** HPV RT-PCR findings of clinically suspected HPV cases and healthy controls HPV: human papillomavirus; RT-PCR: reverse transcription polymerase chain reaction

RT-PCR Results	Cases (n=86), n (%)	Controls (n=63), n (%)
Positive	10 (11.63%)	0
i. HPV-16/31	03 (30%)	-
ii. HPV-18/45	07 (70%)	-
Negative	76 (88.37%)	63 (100%)

Correlation between Pap smear results and HPV RT-PCR results

Among the 86 clinically suspected cases, Pap smear reports were available for 68 cases. None of the cases with normal cytology tested positive for HPV (Table 4). Also, the single case of carcinoma in situ was positive for HPV. Among individuals with inflammatory cytology, a minority (3/22, 13.6%) were HPV positive. HPV positivity was restricted to cytological categories associated with inflammation or high-grade lesions, underscoring the potential of cytological assessment in triaging HPV-related risk.

Among the 18 clinically suspected cases where Pap smear reports were not available, six (33.3%) tested positive for HPV.

**Table 4:** Correlation of Pap smear results with HPV RT-PCR results HPV: human papillomavirus; RT-PCR: reverse transcription polymerase chain reaction

Pap Smear Results		Normal Cytology, n (%)	Inflammatory Cytology, n (%)	Mild Dysplasia, n (%)	Carcinoma In situ, n (%)	Total, n (%)
RT-PCR Positivity	Positive	0	03 (13.64%)	0	01 (100%)	04 (11.63%)
	Negative	44 (100%)	19 (86.36%)	01 (100%)	0	64 (88.37%)
Total		44 (51.16%)	22 (25.58%)	01 (1.16%)	01 (1.16%)	68 (100%)

Nanopore sequencing of full-length 16S rRNA amplicon

Full-length 16S rRNA amplicons were generated as previously described. Following quality filtering, Run 1 yielded 36,19,803 reads, and Run 2 yielded 27,88,418 reads, averaging 72,908.4 and 32,869.5 reads per sample. The mean quality score of sequences in Runs 1 and 2 was 10.1 and 10.3, respectively, and the mean read lengths of the sequences were 1547.2 base pairs (Run 1) and 1569.8 base pairs (Run 2).

On data filtering, out of an initial set of 2,517 OTUs, 905 low-abundance and 57 low-variance features were removed, leaving 512 features for downstream analysis. Singleton features, and those present in only one sample, were excluded to reduce potential artifacts. Data rarefaction to a sequencing depth of 9,365 reads per sample was performed to minimize variability caused by unequal sampling effort. Following the normalization of library sizes, samples with fewer than 10,000 sequencing reads were excluded to minimize potential biases associated with low sequencing depth. As a result, 21 HPV-suspected cases and 17 healthy controls were retained for downstream analysis. The HPV-suspected cases group comprised nine PCR-confirmed HPV-positive cases and 12 cases that were PCR-negative but exhibited inflammatory cytology on Pap smear examination.

Diversity of vaginal microbiota among the three groups

Alpha diversity analysis was performed to assess the richness and diversity of microbial communities across three groups: confirmed HPV infection, healthy individuals, and inflammatory smears. The analysis included Chao1, Abundance-based Coverage Estimator (ACE), Shannon, Simpson, and Fisher indices.

**Figure 1:**
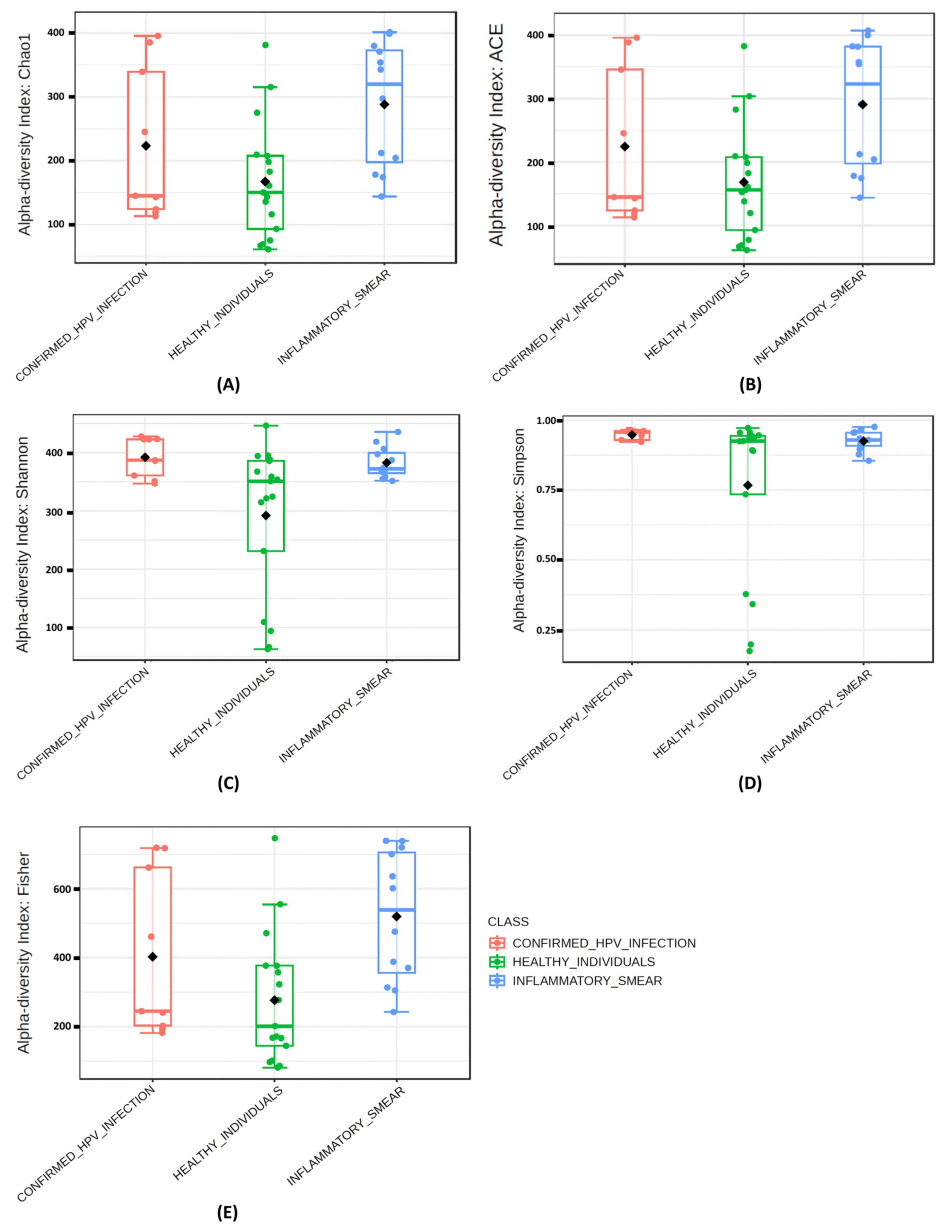
Box Plots Depicting the Alpha Diversity Indices of Vaginal Microbiota among the Three Groups Alpha-diversity Indices estimating species richness (A) Chao1 Index, (B) ACE Index and (E) Fisher Index. Alpha-diversity Indices measuring both species richness and evenness (C) Shannon Index, (D) Simpson Index.

The richness estimators, the Chao1 and ACE indices, showed no significant differences between the confirmed HPV infection group and healthy individuals (*p*-value = 0.24 and *p*-value = 0.25, respectively), nor between the confirmed HPV infection group and the inflammatory smear group (*p*-value = 0.21 and *p*-value = 0.20). However, both indices were significantly higher in the inflammatory smear group than in healthy individuals (*p*-value = 0.003 and *p*-value = 0.003, respectively), suggesting increased richness in inflammatory smears. (Figures 1A, 1B).

Regarding the diversity indices, the Shannon index was significantly higher in both the confirmed HPV infection group and the inflammatory smear group compared to healthy individuals (*p*-value = 0.007 and *p*-value = 0.01, respectively), with no significant difference between the confirmed HPV infection and inflammatory smear groups (*p*-value = 0.49). The Simpson index showed higher diversity in the confirmed HPV infection and inflammatory smear groups than in healthy individuals (*p*-value = 0.02 and *p*-value = 0.04). However, these differences were marginally significant after multiple testing corrections (false discovery rate (FDR) = 0.061). The Fisher index was significantly higher in the inflammatory smear group than in healthy individuals (*p*-value = 0.002), with no significant differences among other comparison groups (Figures 1C-1E).

**Figure 2:**
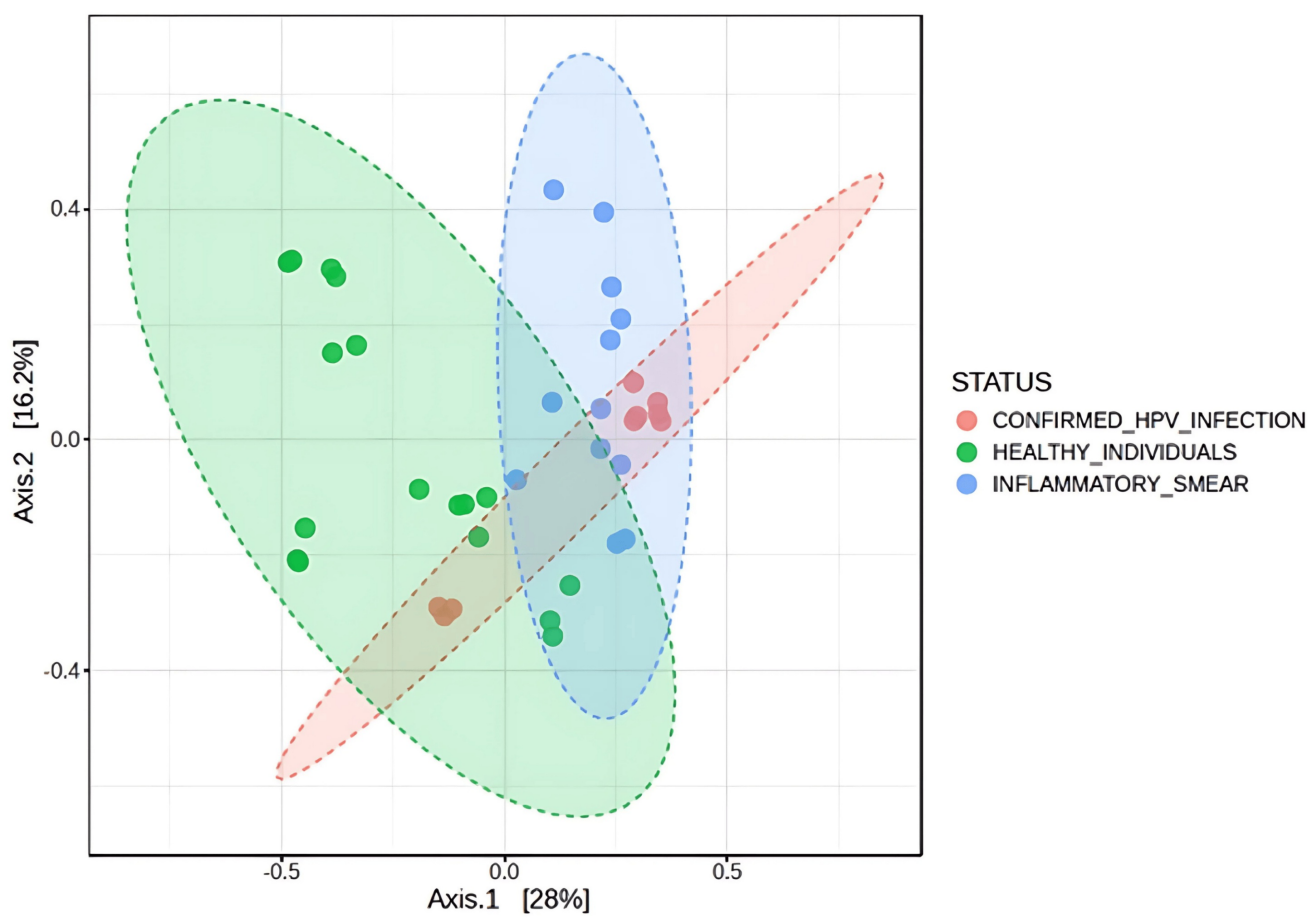
PCoA plot depicting the beta diversity of vaginal microbiota among the three groups PCoA: principal coordinate analysis

Beta diversity analysis using Bray-Curtis dissimilarity was performed to assess differences in microbial community composition among the three groups: confirmed HPV infection, inflammatory smear, and healthy individuals. The overall PERMANOVA test indicated a significant difference in microbial composition across the groups (F = 4.9056, R² = 0.219, *p*-value = 0.001). Pairwise PERMANOVA comparisons confirmed significant differences between all group pairs: confirmed HPV infection versus inflammatory smear (F = 2.97, R² = 0.14, *p*-value = 0.01), confirmed HPV infection versus healthy individuals (F = 4.80, R² = 0.17, *p*-value = 0.001), and inflammatory smear versus healthy individuals (F = 5.89, R² = 0.18, *p*-value = 0.001). In the PCoA plot, samples from healthy individuals clustered separately from those of the disease groups along the first axis, while the second axis differentiated between confirmed HPV infection and inflammatory smear groups (Figure 2).

Taxonomic composition of vaginal microbiota across the three groups

**Figure 3:**
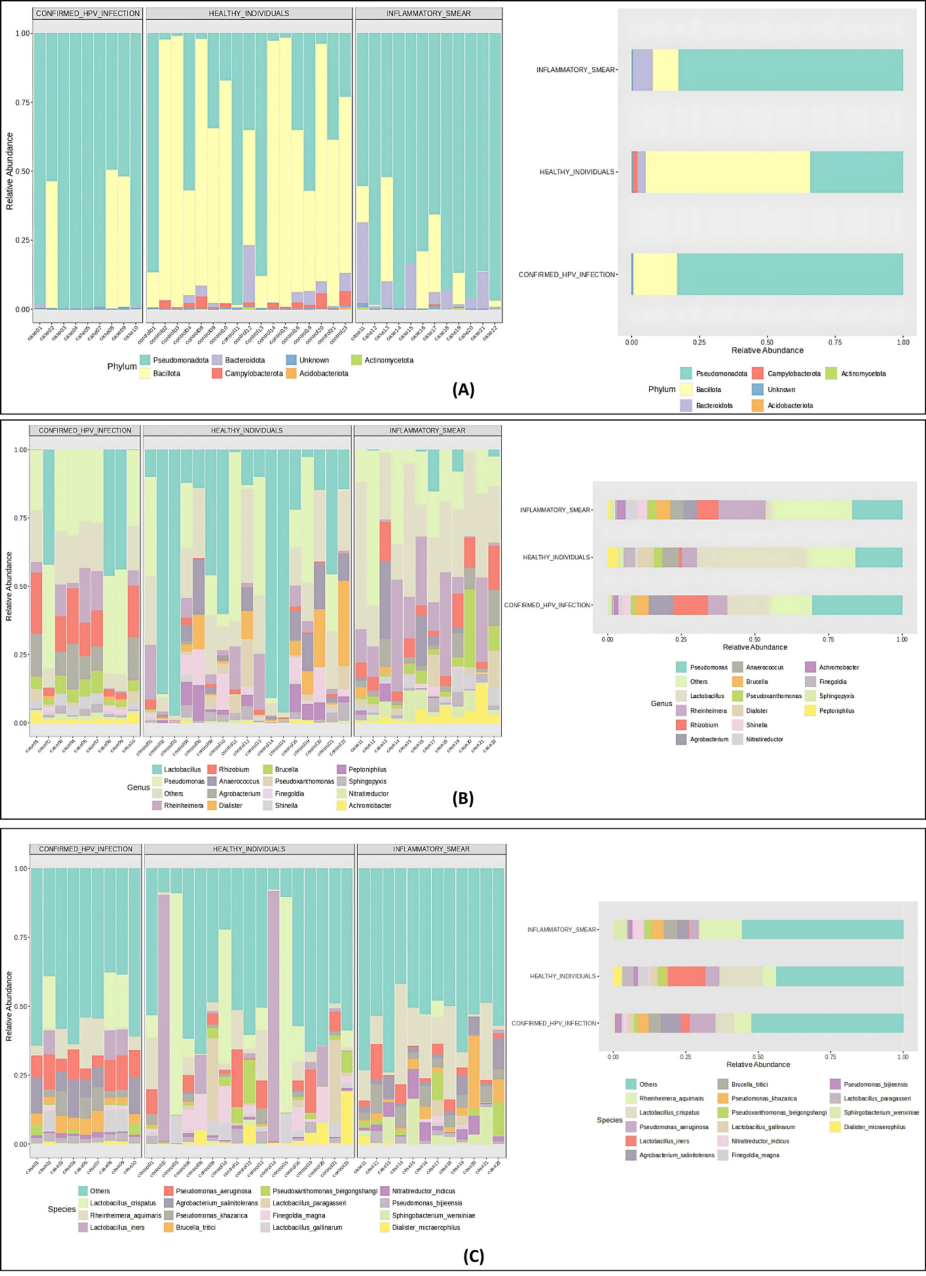
Differential Abundance of Vaginal Microbiota at Phylum, Genus, and Species Levels Across the Three Groups Microbial community composition at (A) phylum level, (B) genus level, and (C) species level, showing sample-wise relative abundance (left image) and grouped relative abundance (right image) across confirmed HPV infection, healthy individuals, and inflammatory smear groups.

Differential abundance analysis revealed distinct microbial compositions across the three groups: confirmed HPV infection, inflammatory smear, and healthy individuals at multiple taxonomic levels (Figure 3). Phylum *Bacillota* was predominant among healthy controls at the phylum level, accounting for approximately 60.79% of the microbial community. Conversely, *Pseudomonadota* showed a marked dominance in the inflammatory smear group (82.60%) and the confirmed HPV-infection group (83.20%).

At the genus level, *Lactobacillus* was the most abundant in healthy individuals, accounting for approximately 37.74% of the microbial community. Other genera included *Pseudomonas* (15.73%), *Anaerococcus* (5.60%), and *Dialister* (5.56%). In the inflammatory smear group, *Pseudomonas* was the predominant genus (17%), followed by *Rheinheimera* (16.06%) and *Rhizobium* (7.01%). For the confirmed HPV infection group, *Pseudomonas* was most dominant (30.60%), followed by *Lactobacillus* (14.76%), *Rhizobium* (11.75%), and *Agrobacterium* (8.41%).

At the species level, healthy individuals were characterized by the abundance of *L. crispatus* (15.06%) and *L. iners* (12.80%). The inflammatory smear group was characterized by the abundance of *Rheinheimera aquimaris* (14.34%), *Brucella tritici* (4.62%), *Pseudomonas khazarica* (4.30%), and *Sphingobacterium wenxiniae* (4.20%). In the confirmed HPV infection group, notable species included *Pseudomonas aeruginosa* (8.58%), *Agrobacterium salintolerans* (6.89%), and *R. aquimaris* (5.98%).

Together, these multi-level taxonomic findings highlight the reduction in *Lactobacillus* abundance in disease states, particularly at genus and species levels, indicating a loss of beneficial microbes often associated with vaginal health. The dominance of *Pseudomonadota* and associated genera/species in disease groups contributes to dysbiosis and suggests a microbial shift linked to inflammatory or infectious conditions.

Classification of vaginal microbiota into CSTs

Based on the dominant *Lactobacillus* species, the samples were classified into different CSTs using the VALENCIA algorithm and CST architecture. In recent taxonomic revisions of the genus *Lactobacillus*,* Lactobacillus paragasseri* has been recognized as a distinct species, separated from *Lactobacillus gasseri* based on genomic and phylogenetic analyses [13]. Since the dominance of *L. gasseri* defines CST II, samples exhibiting dominance of the closely related sister taxon *L. paragasseri* were also categorized under CST II. The distribution of CSTs observed in the present study is summarized in Table 5.

**Table 5:** Vaginal CSTs among HPV-suspected cases and healthy controls *p-*values were calculated using two-tailed Fisher’s exact test CST: community state type; HPV: human papillomavirus

CST		HPV-suspected Cases, n (%)	Healthy Controls, n (%)	Grand Total, n (%)	*p*-value
I	I-B (less *L. crispatus* but still majority)	3 (14.29%)	7 (41.18%)	10 (26.32%)	0.078
II	II (dominated by *L. gasseri*)	6 (28.57%)	4 (23.53%)	10 (26.32%)	1.00
III	III-A (almost completely *L. iners*)	0 (0%)	2 (11.76%)	2 (5.26%)	1.00
	III-B (less *L. iners* but still majority)	2 (9.52%)	0 (0%)	2 (5.26%)	
IV	IV-B (high to moderate relative abundance of *G. vaginalis* and *A. vaginae*)	1 (4.76%)	0 (0%)	1 (2.63%)	0.307
	IV-C0 (relatively even community with *Prevotella* species)	4 (19.05%)	4 (23.53%)	8 (21.05%)	
	IV-C1 (dominated by *Streptococcus* species)	1 (4.76%)	0 (0%)	1 (2.63%)	
	IV-C2 (dominated by *Enterococcus* species)	2 (9.52%)	0 (0%)	2 (5.26%)	
	IV-C4 (dominated by *Staphylococcus* species)	1 (4.76%)	0 (0%)	1 (2.63%)	
V	V (dominated by *L. jensenii*)	1 (4.76%)	0 (0%)	1 (2.63%)	-
Grand Total		21 (100%)	17 (100%)	38 (100%)	

**Figure 4:**
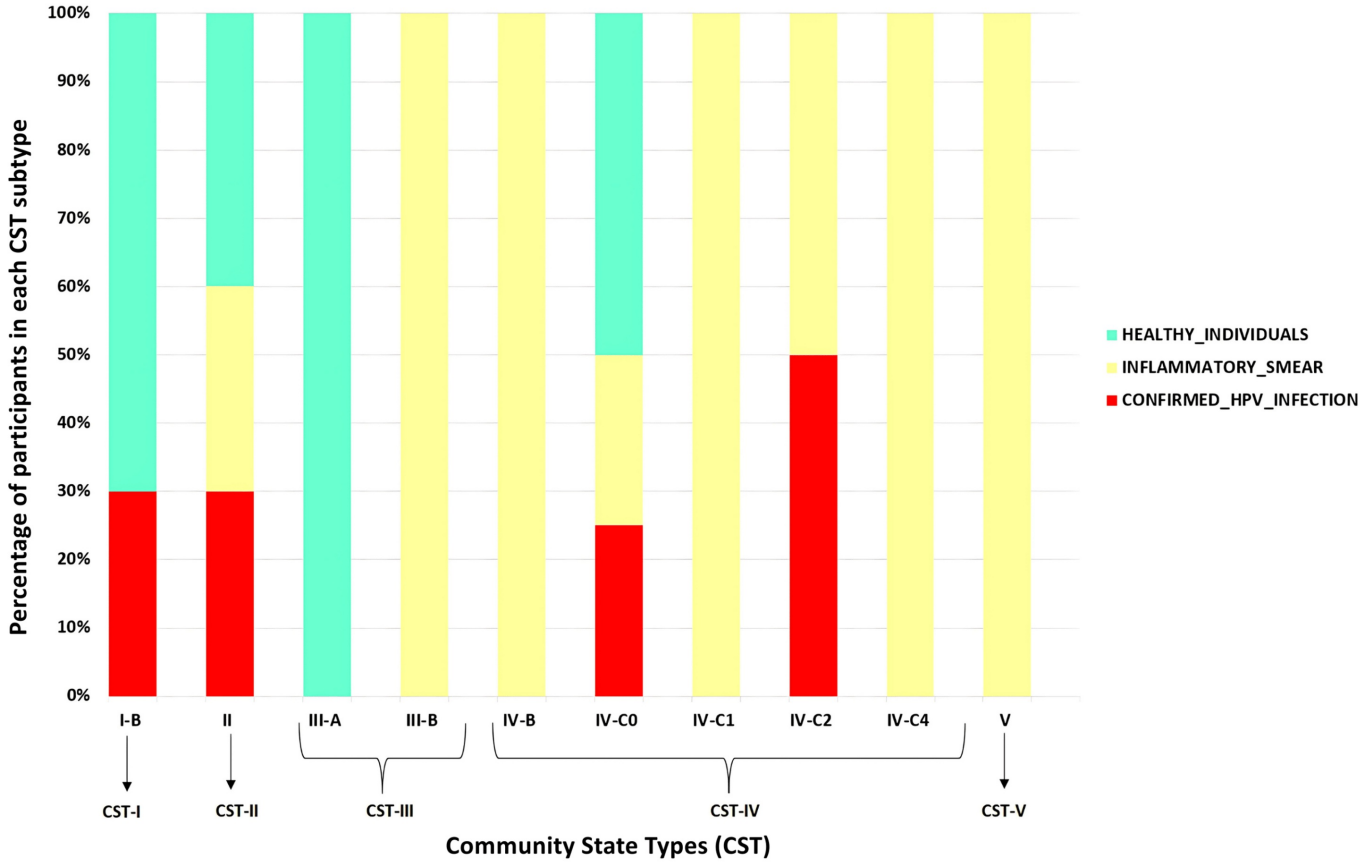
Distribution of vaginal CSTs among the three groups CST: community state type

CST I, typically dominated by *L. crispatus*, was more common in healthy controls (7/17, 41.2%) than in suspected cases (3/21, 14.3%). In contrast, CST IV associated with higher microbial diversity and anaerobic taxa was predominant among suspected cases (9/21, 47.6%) compared to controls (4/17, 23.5%). Other CSTs, including CST II and CST III, were similarly distributed across groups, with no significant differences observed. These findings suggest a possible shift from protective to dysbiotic community types in HPV-infected cases (Figure 4).

Potential bacterial biomarker using linear discriminant analysis effect size (LEfSe) analysis

**Figure 5:**
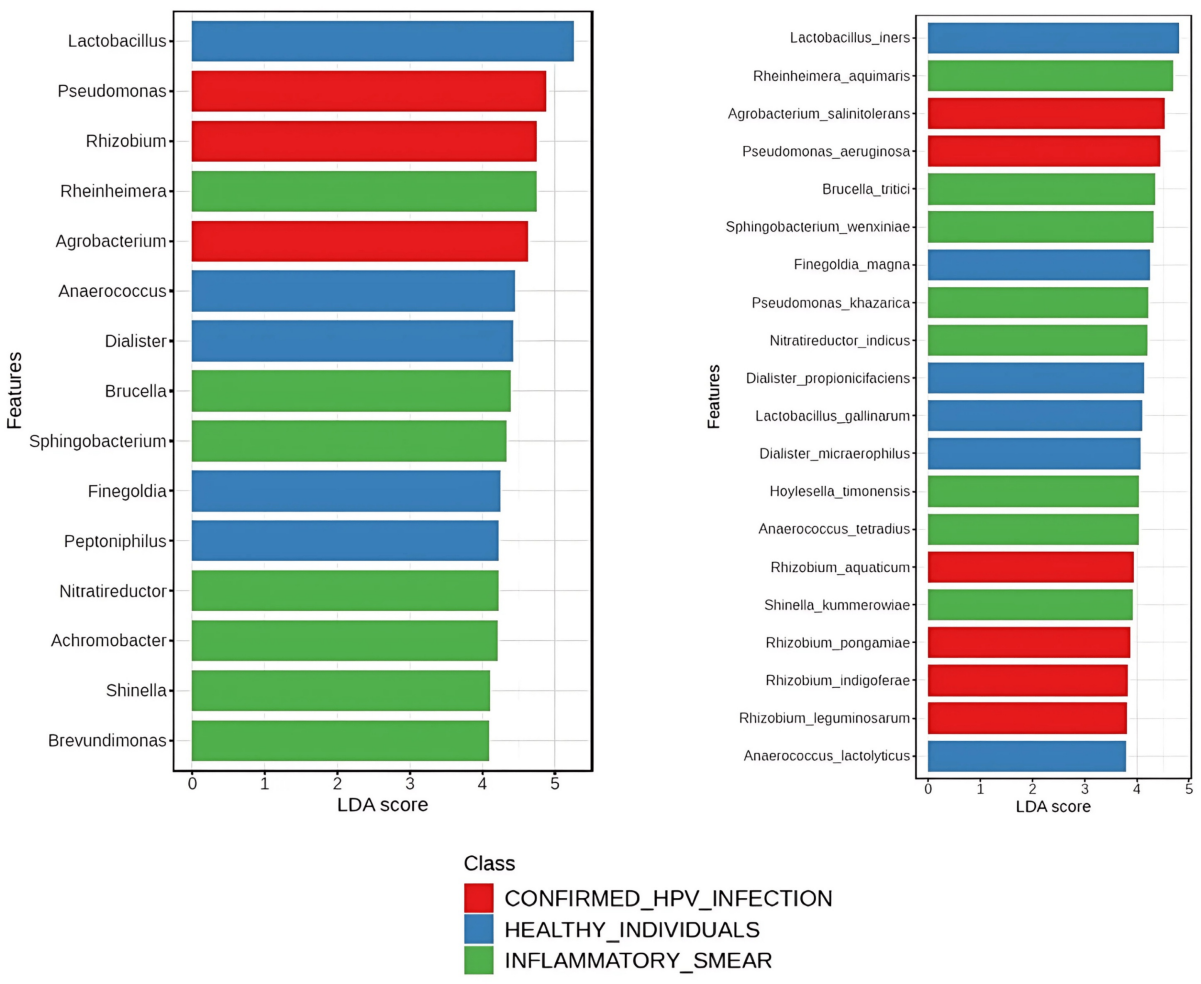
Microbial biomarkers identified by LEfSe in vaginal microbiota of the three groups LEfSe: linear discriminant analysis effect size; LDA: linear discriminant analysis

LEfSe analysis was performed to identify microbial taxa that are differentially abundant between the three groups: confirmed HPV infection, inflammatory smear, and healthy individuals. The study revealed significant differences at multiple taxonomic levels (Figure 5).

At the genus level, 111 genera were found to be significantly different between the groups based on linear discriminant analysis (LDA) score threshold. Genus *Lactobacillus*, *Anaerococcus*, *Dialister*, *Finegoldia*, and *Peptinophilus* were strongly linked to healthy controls, suggesting their role in maintaining a balanced vaginal microbiome. While genera including *Rheinheimera*, *Brucella*, *Sphingobacterium*, *Nitratireductor*, *Achromobacter*, *Shinella*, and *Brevundimonas* were linked to the inflammatory smear group, genera *Pseudomonas*, *Rhizobium*, and *Agrobacterium* were strongly related to the confirmed HPV-infected group.

Similarly, 271 species demonstrated significant differences between the study groups at the species level. *L. iners*, *Finegoldia magna*, *Dialister propionicifaciens*, *Lactobacillus gallinarum*, and *Dialister micraerophilus* exhibited strong positive associations with healthy controls, suggesting their beneficial roles in maintaining vaginal microbiome health. Conversely, *R. aquimaris*, *Brucella tritici*, *Sphingobacterium wenxiniae*, *Pseudomonas khazarica*, and *Nitratireductor indicus* were strongly associated with the inflammatory smear group. Additionally, *Agrobacterium salinitolerans*, *P. aeruginosa*, *Rhizobium aquaticum*, *Rhizobium pongamiae*, and *Rhizobium indigoferae* exhibited significant associations with the HPV-positive group.

Thus, LEfSe analysis identified key genera and species potentially important for microbial balance or dysbiosis, providing insights into microbial biomarkers that could aid in diagnosing or monitoring vaginal health.

## Discussion

India has an estimated 511.4 million women aged 15 years and older who are at risk for cervical cancer. It is the second most common cancer among women in India, and it also ranks second among women aged between 15 and 44 years. Approximately 5% of women in the general population carry cervical HPV-16/18 infection at any given time, with HPV types 16 or 18 responsible for 83.2% of invasive cervical cancer cases [14]. The overall prevalence of HPV infection in the study population (including cases and controls) was 6.71% among women aged between 20-50 years, which is concordant with the studies from India suggesting HPV prevalence ranging from 2.3% to 36.9%. Some studies found that the frequency of HPV varied greatly throughout India, from 4.7% in Kolkata to 6.1% in the south to 19.2% among indigenous women in central India [15]. Similarly, another study reported that population-based estimates of HPV prevalence among women in the general population in India range from 7.5% to 16.9% [16]. While HPV 18/45 was the predominant HPV genotype detected in the present study, previous studies from South Andaman, Central India, Madhya Pradesh, Tamil Nadu, and Andhra Pradesh have shown HPV 16 as the most prevalent genotype [15].

Cervical cancer is a multifactorial disease influenced by host, microbial, and environmental factors. While HPV remains the primary established risk factor, dysbiosis of the cervicovaginal microbiome has increasingly been recognized as critical in HPV infection, inflammation, persistence, and cervical carcinogenesis [17]. Consistent with previous findings, our study demonstrated that women with inflammatory smears and confirmed HPV infection had higher vaginal microbial diversity and distinct bacterial taxa compared to healthy controls [18-20]. Specifically, we observed a reduced abundance of *Lactobacillus* in women with dysbiotic vaginal microbiota.

*Lactobacillus *species dominate a healthy vaginal microbiome, thriving anaerobically and producing lactic acid, hydrogen peroxide (H_2_O_2_), and bacteriocins, which collectively protect against pathogenic invasion. These bacteria primarily generate L- and D-lactic acids, maintaining vaginal pH below 4.5, with vaginal epithelial cells contributing approximately 20% of L-lactic acid. Although hydrogen peroxide has antimicrobial properties, its physiological relevance is debated due to its limited effectiveness at physiological concentrations and potential self-toxicity at higher levels. *Lactobacillus* also produces bacteriocins, antimicrobial peptides that disrupt membranes of non-native microbes, and competes effectively for epithelial binding sites, preventing pathogen adhesion [7,20,21]. Disruption of this microbial balance, termed dysbiosis, promotes cervical carcinogenesis by compromising the epithelial barrier, inducing metabolic dysregulation, abnormal cell proliferation, genomic instability, chronic inflammation, and angiogenesis. Thus, *Lactobacillus* species play a pivotal role in maintaining cervical epithelial integrity and inhibiting HPV entry into basal keratinocytes [21].

In our study, healthy individuals were characterized by dominance of *L. crispatus* and *L. iners*. Although the variation in dominant *Lactobacillus* species across different ethnic groups is well-documented [21], it is noteworthy that even within the Indian population, regional differences in the prevalent *Lactobacillus* species are observed. While studies from western and northern India have reported *L. iners* as the predominant species in healthy vaginal flora [22-24], a study from the northeastern region has identified *Lactobacillus mucosae* as the most prevalent *Lactobacillus* species among northeastern women [25]. These findings highlight the potential influence of geographic, ethnic, and environmental factors on vaginal microbiota composition, underscoring the need for region-specific microbial reference data in understanding disease associations.

The vaginal microbiome produces two lactic acid isomers: L-lactic acid (by vaginal epithelium, *L. iners*, and anaerobic bacteria) and D-lactic acid (predominantly by *L. jensenii*, with both isomers produced by *L. crispatus* and *L. gasseri*). High concentrations of D-lactic acid, notably from *L. crispatus*-dominated microbiota, enhance vaginal mucus viscosity, trapping viral particles effectively. Thus, CST-I (*L. crispatus*) and CST-II (*L. gasseri*) are typically found in HPV-negative women, with *L. gasseri* notably aiding rapid HPV clearance [21]. In the present study as well, healthy individuals were predominantly associated with CST-I, reinforcing its role as an optimal vaginal community state. Conversely, CST-III and CST-IV are associated with dysbiosis. In line with previous findings, our study also found that HPV-infected women were predominantly associated with CST-IV. Microbial communities corresponding to CST-III and CST-IV are known to exhibit elevated L- to D-lactic acid ratios, which can upregulate the expression of extracellular matrix metalloproteinase inducer (EMMPRIN) and matrix metalloproteinase-8 (MMP-8). This enzymatic activity leads to collagen degradation and compromises cervical tissue integrity, thereby facilitating HPV entry and potentially contributing to cancer progression [21].

Literature states that women with CST-IV microbiota exhibit elevated levels of pro-inflammatory cytokines, including tumor necrosis factor alpha (TNF-α), IL-1α, interleukin (IL)-1, interferon gamma (IFN-γ), IL-4, IL-10, IL-12, and IL-8, compared to those with CST-I. Also, CST-III was associated with increased IFN-γ levels. Longitudinal studies have further demonstrated rising TNF-α, IL-1α, and IL-1β concentrations in individuals transitioning from CST-I or III to CST-IV, suggesting a shift toward a pro-inflammatory state. In contrast, CST-I and V, typically dominated by *L. crispatus* and *L. jensenii*, induced minimal cytokine responses in reconstructed vaginal epithelial models, highlighting the protective role of *Lactobacillus*-dominant communities [26]. Building on this understanding of microbiota-immune interactions, a pilot study evaluating the use of probiotics containing *Lactobacillus casei* in women with HPV-associated cervical abnormalities saw greater clearance of cytological abnormalities and HPV among probiotic users compared to controls. These findings suggest that modulating the vaginal microbiota through probiotics may enhance immune responses and support the resolution of HPV-related cervical lesions [27].

Most studies have reported that in individuals with vaginosis or dysbiosis, there is a sharp decline or loss of *Lactobacilli* and a corresponding increase in the concentration of facultative or obligate anaerobic microbes, such as *Gardnerella*, *Prevotella*, *Atopobium*, *Mobiluncus*, *Bifidobacterium*, *Sneathia*, *Leptotrichia*, *Peptostreptococcus*, *Fusobacterium*, and some novel bacteria in *Clostridiales*. These organisms cause dysbiosis primarily by producing virulence factors such as sialidases, vaginolysin, and other cytotoxins, degrading protective mucus barriers, damaging epithelial integrity, and facilitating pathogen entry. Certain bacteria, like *Peptostreptococcus anaerobius *and *Fusobacterium nucleatum*, promote dysbiosis by inducing inflammation and tumorigenesis [28]. In contrast to previous findings, the present study revealed that a significant reduction in *Lactobacilli* among HPV-infected participants was associated with an increased presence of bacterial genera commonly associated with soil and aquatic environments, such as *Rhizobium*, *Rheinheimera*, *Agrobacterium*, *Brucella*, *Achromobacter*, *Shinella*, and *Nitratireductor*. The roles of these environmental bacteria in vaginal dysbiosis and their potential involvement in HPV infection remain poorly understood and warrant further investigation. Supporting this observation, *Acidovorax* and *Oceanobacillus*, also primarily environmental bacteria, have previously been linked with HPV infection among pregnant Chinese women [29]. The presence of these environmental bacteria in vaginal flora could be linked to hygiene practices. For instance, washing or douching using water from contaminated sources, such as groundwater, surface water, or soil-contaminated water, could introduce soil- or water-associated bacteria into the vaginal environment. Additionally, personal hygiene products, particularly if improperly stored or contaminated, might serve as a source for these bacteria. Occupational and lifestyle factors, such as agricultural work or frequent contact with soil and water, may also increase exposure to these environmental organisms. This notion is supported by findings from a study demonstrating that the vaginal microbiome composition varies significantly with water source, type of sanitation facility, rainfall, and hygiene-related behaviors, even after adjusting for demographic and behavioral variables. These findings suggest that environmental exposure may play a more crucial role in shaping the vaginal microbial community than previously understood [30]. Furthermore, certain bacteria identified in this study, including *Achromobacter* [31], *Brucella* [32], and occasionally *Agrobacterium* [33,34], have previously been recognized as opportunistic pathogens and have been shown to cause genitourinary infections and other infections in the immunocompromised host. Thus, the potential influence of environmentally derived microbes on vaginal microbiome dynamics and HPV pathogenesis represents an interesting topic for future research.

Limitations of the study

This study had several limitations. First, the HPV detection kit did not comprehensively test for all high-risk and low-risk HPV types. As a result, some HPV infections, especially in the control group, may have gone undetected, thereby underestimating the true prevalence of HPV infection in the study population. Specifically, the test did not differentiate between individual genotypes within pooled groups such as HPV 16/31 and HPV 18/45, limiting the ability to identify genotype-specific associations with vaginal microbiota composition. Secondly, microbiome analysis was performed on a subset of participants who met quality thresholds for sequencing depth. This further reduced the effective sample size and potentially limited the statistical power for detecting subtle microbial differences. Thirdly, the study did not collect data on the socioeconomic status, the area of residence (urban or rural), or the hygiene practices of participants. This was intentionally excluded to avoid selection bias; however, these factors are known to influence the vaginal microbiota composition and HPV prevalence. Finally, due to the study's cross-sectional design, it was not possible to establish the causal relationship between HPV infection, microbiota alterations, and inflammation.

## Conclusions

The present study revealed distinct microbiological characteristics distinguishing women with suspected HPV infection from healthy controls. HPV-18/45 emerged as the predominant genotype, identified in nearly one-fourth of the cases. Vaginal microbiome profiling demonstrated increased microbial diversity, a notable reduction in *Lactobacilli* abundance, and enrichment of opportunistic taxa among symptomatic women. The higher prevalence of CST-IV in this group further indicated a shift toward vaginal dysbiosis. LEfSe analysis identified key discriminatory taxa across taxonomic levels. Together, these findings highlight the potential influence of vaginal microbiota on HPV persistence and suggest specific microbial signatures as candidate biomarkers for cervical disease risk assessment and targeted microbiome-based interventions.
